# Investigating placental disorders of pregnancy in sub-Saharan Africa: addressing the gap in a neglected area of research

**DOI:** 10.1186/s12978-020-0871-x

**Published:** 2020-04-30

**Authors:** France Donnay, Mai-Lei Woo Kinshella, Meriel Flint-O’Kane, Peter von Dadelszen, Laura Magee, Marianne Vidler

**Affiliations:** 1 Department of Women and Children’s Health, School of Life Course Science, Faculty of Life Sciences and Medicine, King’s College London, 5th Floor, Becket House, 1 Lambeth Palace Road, London, SE1 7EU UK; 2 Department of Obstetrics and Gynecology, Faculty of Medicine, University of British Columbia, Vancouver, Canada

The widely-cited failure within the Millennium Development Goals to adequately reduce infant deaths and reverse maternal health inequalities prompted incorporation of new goals in the 2016 UN Sustainable Development Goal (SDG) three to ‘ensure healthy lives and promote well-being for all at all ages’ [1–4]. With ambitious goals to reduce the global mortality ratio to less than 70 per 100,000 live births and neonatal mortality to less than 12 per 1000 live births, we need to accelerate cross-disciplinary discovery and a robust research platform to address these targets. The PRECISE Network seeks to plays its role by focusing on the placental disorders.

Annually, the placental disorders of pregnancy (pregnancy hypertension, fetal growth restriction and stillbirth) are associated with 46,000 maternal and 2.5 million perinatal deaths [5]. Over 99% of these deaths occur in low- and middle-income countries (LMICs) and over half in sub-Saharan Africa [5, 6]. In our opinion, this disparity in outcomes between LMICs and high-income countries (HICs) represents a global injustice.

While placental disorders of pregnancy are well investigated in HICs, this is not so in LMICs. LMIC-based placental disorder research is under-represented in the global portfolio. Moreover, the pathways to disease within a resource-constrained setting in sub-Saharan Africa are likely to differ from pathways in HICs where much of the research has been conducted. For example, synergistic challenges of malnutrition, rural settings underserved by transport infrastructure and health facilities, high burdens of infectious diseases, and other adverse social determinants of health may contribute to pathways of disease in ways that are currently not captured by HIC research. Understanding the interactions between placental complications and contextual factors may provide a critical step towards the development of precision healthcare to optimise maternal, fetal and infant outcomes.

The PRECISE (PREgnancy Care Integrating translational Science, Everywhere) Network is a new and interdisciplinary group of research scientists and health advocates from sub-Saharan Africa, Europe, Canada and the USA, that is investigating the origins and outcomes of the placental disorders in sub-Saharan Africa. Funded by the UK Research and Innovation Global Challenges Research Fund (GCRF), PRECISE aims to develop a robust clinic-based research infrastructure, database and biobank that will catalyse formative research describe the pathways to placental disorders and their complications in sub-Saharan Africa, and inform the design of effective interventions. Importantly, this programme will provide essential information about the impact of placental disorders on women’s lives and their communities.

The five papers in this supplement introduce the scientific scope and vision of this ambitious programme of work and describe the development of key components of PRECISE: the study protocol, clinical database, biorepository, qualitative work plans, and partnership and capacity-building elements. The first three papers describe the design of the prospective pregnancy cohort study and the research platform to collect epidemiological data and biological samples from approximately 10,000 pregnant Gambian, Kenyan and Mozambican women, 1500 of whom we anticipate will have a pregnancy complicated by at least one placental disorder. In addition, we will recruit 1800 non-pregnant women of reproductive age as a control group to provide representative regional data and biological samples. The unified data collection platform of the PRECISE database and the biorepository links together sociological, clinical and discovery bioscience research. The second and third papers in this series describe the issues and challenges around their creation, development, management and scientific uses. PRECISE takes a 360° approach to understanding both individual-level co-exposures and contextual factors associated with the placental disorders. The fourth paper in the series describes the value of qualitative research in helping to understand quantitative and biological data. The fifth paper in the series brings the picture of the PRECISE Network together with capacity-building in research infrastructure, strengthening emerging leaders in reproductive science and supporting advocacy for research. Together, the papers in this supplement describe a vision and the developmental journey of research platform that seeks to fill current knowledge gaps related to placental conditions in sub-Saharan Africa, and strengthens capacity for research and health improvement related to placental disorders.
